# Sildenafil improves radiation‐induced oral mucositis by attenuating oxidative stress, NF‐κB, ERK and JNK signalling pathways

**DOI:** 10.1111/jcmm.17480

**Published:** 2022-07-10

**Authors:** Moein Ala, Razieh Mohammad Jafari, Mahan Ala, Sedigheh Marjaneh Hejazi, Seyed Mohammad Tavangar, Seied Rabi Mahdavi, Ahmad Reza Dehpour

**Affiliations:** ^1^ Experimental Medicine Research Center, School of medicine Tehran University of Medical Sciences Tehran Iran; ^2^ Department of Pharmacology, School of Medicine Tehran University of Medical Sciences Tehran Iran; ^3^ Faculty of Dentistry Golestan University of Medical Sciences Gorgan Iran; ^4^ Medical Physics and Biomedical Engineering Department, School of Medicine Tehran University of Medical Sciences Tehran Iran; ^5^ Advanced Medical Technologies and Equipment Institute Research Center for Molecular and Cellular in Imaging, Bio‐optical Imaging Group Imam Khomeini Hospital, Tehran University of Medical Sciences Tehran Iran; ^6^ Department of Pathology, Shariati Hospital Tehran University of Medical Sciences Tehran Iran; ^7^ Chronic Diseases Research Center, Endocrinology and Metabolism Population Science Institute Tehran University of Medical Sciences Iran; ^8^ Radiation Biology Research Center Iran University of Medical Sciences Tehran Iran; ^9^ Department of Medical Physics, School of medicine Iran University of Medical Sciences Tehran Iran

**Keywords:** apoptosis, inflammation, oxidative stress, radiation‐induced oral mucositis, sildenafil

## Abstract

Radiation‐induced oral mucositis is a common and dose‐limiting complication of head and neck radiotherapy with no effective treatment. Previous studies revealed that sildenafil, a phosphodiesterase 5 inhibitor, has anti‐inflammatory and anti‐cancer effects. In this study, we investigated the effect of sildenafil on radiation‐induced mucositis in rats. Two doses of radiation (8 and 26 Gy X‐ray) were used to induce low‐grade and high‐grade oral mucositis, separately. A control group and three groups of sildenafil citrate‐treated rats (5, 10, and 40 mg/kg/day) were used for each dose of radiation. Radiation increased MDA and activated NF‐κB, ERK and JNK signalling pathways. Sildenafil significantly decreased MDA level, nitric oxide (NO) level, IL1β, IL6 and TNF‐α. The most effective dose of sildenafil was 40 mg/kg/day in this study. Sildenafil also significantly inhibited NF‐κB, ERK and JNK signalling pathways and increased bcl2/bax ratio. In addition, high‐dose radiation severely destructed the mucosal layer in histopathology and led to mucosal cell apoptosis in the TUNEL assay. Sildenafil significantly improved mucosal structure and decreased inflammatory cell infiltration after exposure to high‐dose radiation and reduced apoptosis in the TUNEL assay. These findings show that sildenafil can improve radiation‐induced oral mucositis and decrease the apoptosis of mucosal cells via attenuation of inflammation and oxidative stress.

## 1 INTRODUCTION

Oral mucositis is an acute inflammatory disease with specific causes. Radiation‐induced oral mucositis is a dose‐limiting complication of head and neck cancer radiotherapy. Radiation‐induced oral mucositis happens in more than 80% of patients with head and neck cancer who undergo radiotherapy. Female sex, older age, malnutrition, smoking, chronic kidney disease, poor oral hygiene and decreased secretion of salvia are associated with a higher prevalence of radiotherapy‐induced oral mucositis.^1^ Five stages have been proposed in the development of radiation‐induced oral mucositis including 1—Initiation of tissue injury and release of reactive oxygen species (ROS); 2—Relaying inflammatory signal and amplification of inflammation; 3—Breakage of the mucosal barrier by an intensive inflammatory response; 4—Ulceration and bacterial infection; and 5—Healing and proliferation.^2^, ^3^

However, numerous studies have attempted to introduce an effective remedy for oral mucositis; still, there is not a consensus regarding the management of oral mucositis and current treatment protocols focus on symptomatic management.^2^ High‐dose radiation leads to the release of a large amount of ROS and inflammatory cytokines and the production of pro‐apoptotic proteins which endangers mucosal integrity.^4^ Therefore, several groups of drugs with different mechanisms of action are presumed to mitigate the inflammatory response or contribute to the recovery of the mucosal barrier. In this regard, growth factors, immunosuppressive agents, anti‐oxidants and even herbal drugs with anti‐inflammatory properties were investigated in animal models and clinical studies.^5^ Recently, a genome‐wide study has shown that perturbation of JNK‐related stress can increase the susceptibility to radiation‐induced oral mucositis.^6^ Similarly, several studies have shown that attenuating impaired and uncontrolled NF‐κB signalling can partly alleviate the inflammatory phase of oral mucositis.^7^, ^8^ JNK and NF‐κB activation disrupts mucosal cells tight junction and endangers mucosal integrity.^9^ Stimulation of ERK may be followed by an acceleration of the healing phase in the oral mucositis, but can also be associated with the proliferation of neutrophils and macrophages and a more severe inflammatory response.^10^, ^11^

Sildenafil is a phosphodiesterase 5 inhibitor and a vasodilator potentiating the nitric oxide system. It is commonly used in erectile dysfunction and pulmonary arterial hypertension.^12^ Recently, it has been shown that the drug can improve several inflammatory and non‐inflammatory diseases other than its conventional indications.^12^ Phosphodiesterase 5 inhibition can alleviate inflammation and downregulate NF‐κB signalling pathway.^13^ In addition to its suppressive effect on NF‐κB, sildenafil can attenuate JNK‐ and ERK‐mediated inflammation, apoptosis and cell injury.^14^ Sildenafil increases the bioavailability of cyclic guanosine monophosphate (cGMP) to suppress inflammation.^15^ Sildenafil also enhances the healing process.^16^ The drug can partly attenuate oxidative stress, prevent inflammatory cytokines release and protect against apoptosis.^12^ In addition, sildenafil can protect endothelial cells against radiation‐induced oxidative stress.^17^ Interestingly, numerous studies have shown that sildenafil possesses anti‐tumour properties that can be useful when added to radiotherapy.^12^, ^18^, ^19^

Because of its protective effects against oxidative stress and inflammation, we hypothesized that sildenafil citrate might ameliorate radiation‐induced oral mucositis.

## 2 MATERIALS AND METHODS

### 2.1 Animals and grouping

Forty‐five male Wistar rats weighing 180–220 g were used in this study. Animals were provided by the Department of Pharmacology, Faculty of Medicine, Tehran University of Medical Sciences (TUMS). They were housed in a temperature‐controlled room (25 ± 2°C) with free access to food and water. We anaesthetised rats with ketamine (87 mg/kg) and xylazine (13 mg/kg) before irradiation and tissue sample collection.^20^ They were killed with a CO_2_ chamber at the end of the study.

The effect of sildenafil was measured on both low‐dose (8 Gy) and high‐dose (26 Gy) radiation‐induced mucositis. Sildenafil citrate was dissolved in normal saline, and three doses of sildenafil citrate 5, 10 and 40 mg/kg/day were administered intraperitoneally in this study. These doses of sildenafil are usually used in rats and do not show significant adverse effects.^21^, ^22^

Rats were divided into 9 groups including one healthy control group (intact) and 8 irradiated groups as follows: low‐dose X‐ray control group receiving 8 Gy X‐ray + normal saline, high‐dose X‐ray control group receiving 26 Gy X‐ray + normal saline, sildenafil citrate 5 mg/kg/day +8 Gy X‐ray, sildenafil citrate 5 mg/kg/day +26 Gy X‐ray, sildenafil citrate 10 mg/kg/day +8 Gy X‐ray, sildenafil citrate 10 mg/kg/day +26 Gy X‐ray, sildenafil citrate 40 mg/kg/day +8 Gy X‐ray and sildenafil citrate 40 mg/kg/day +26 Gy X‐ray (Table 1). Each group consisted of 5 rats.

**TABLE 1:** Animals grouping after irradiation

Low‐dose irradiation	High‐dose irradiation
1) Normal saline +8 Gy X‐ray (low‐dose X‐ray control group)	5) Normal saline +26 Gy X‐ray (high‐dose X‐ray control group)
2) Sildenafil citrate 5 mg/kg/day +8 Gy X‐ray	6) Sildenafil citrate 5 mg/kg/day +26 Gy X‐ray
3) Sildenafil citrate 10 mg/kg/day +8 Gy X‐ray	7) Sildenafil citrate 10 mg/kg/day +26 Gy X‐ray
4) Sildenafil citrate 40 mg/kg/day +8 Gy X‐ray	8) Sildenafil citrate 40 mg/kg/day +26 Gy X‐ray

Treatment with sildenafil or normal saline started 1 day before radiotherapy and animals were treated with a daily dose of them for 4 days. Animals were followed and their tongues specimens were collected 7 days after irradiation. Haematoxylin and eosin (H&E) staining and molecular assessments such as Western blotting, and enzyme‐linked immunosorbent assay (ELISA) were performed in low‐dose groups. Furthermore, H&E staining and TUNEL assay were performed in high‐dose groups.

### 2.2 Ethics statement

All experiments were conducted following the Pharmacology department of Health Guide for the Care and Use of Laboratory Animals. The experimental procedures were approved by the Animal Research Committee of Tehran University of Medical Sciences (IR.TUMS.MEDICINE.REC.1399.138).

### 2.3 Radiation protocol for inducing oral mucositis

We performed irradiation in a single session. The head and neck of rats (above their upper extremities) were exposed to radiation. They received an accumulating dose of 8 Gy (4 groups) or 26 Gy (4 groups) using a conventional high‐energy linear accelerator (6MV Elekta Compact Energy), at a dose rate of 2 Gy/minute, SSD (source to surface distance) 101 cm.^4^ Rats were placed side‐by‐side in the prone position along the borders of a 40 cm × 40 cm square cone. Four groups of rats (low‐dose groups or high‐dose groups) were irradiated all at the same time, and their heads were placed on the isocenter plane of the device.

### 2.4 Histopathology

H&E staining was used to determine the severity of mucosal damage and measure the extent of epithelial defects, distortion of the normal mucosal structure and inflammatory cells infiltration. Furthermore, we used histopathological grading criteria for oral mucositis, as proposed by Sunavala‐Dossabhoy et al.^23^ (Table 2). Seven days after irradiation, rats were anaesthetised and their tongues were cut. Tongue tissues were fixed in formaldehyde 4%. After the fixation process, slices with 5 μm thickness were prepared. Each slice was stained with H&E. An expert pathologist blinded to the samples interpreted them.

**TABLE 2:** Histopathological criteria for staging of oral mucositis (Previously proposed by Sunavala‐Dossabhoy et al.^23^)

Grade	Histopathological manifestation
0	Normal mucosa.
1	Focal or diffuse alteration of basal cell layer with nuclear atypia and ≤2 dyskeratotic squamous cells.
2	Epithelial thinning (2–4 cell layer) and/or ≥3 dyskeratotic squamous cells in the epithelium.
3	Loss of epithelium without a break in keratinization or presence of atrophied eosinophilic epithelium.
4	Sub‐epithelial vesicle or bullous formation.
5	Complete loss of epithelial and keratinized cell layers; ulceration.

### 2.5 TUNEL assay

TUNEL assay was performed to measure the effect of radiation and sildenafil on DNA fragmentation and apoptosis of mucosal cells.

The TUNEL assay was done using In Situ Cell Death Detection Kit, POD; Germany, Roche. Tissue sections were deparaffinized and incubated in xylol for 10 min at room temperature. Thereafter, samples were immersed in ethanol 90%, 80% and 70%, consecutively. The slides were rinsed with PBS. Tissue sections were incubated with permeabilization solution (0.3% Triton) for 30 min, and then, slides were washed with PBS, again. Then, slides were incubated in diluted (1:1500) proteinase K solution for 10 min at 37°C. Samples were washed with PBS two times. Then, 50 μl of the TUNEL staining TdT enzyme was added to each slide to cover the tissue section. Samples were incubated in a water bath for 1 h at 37°C and protected from light exposure. POD was added and then 3,3′‐diaminobenzidine (DAB) kit was used and then samples were washed with PBS. DAB solution was added to samples and washed with water after 3–5 min. Then, haematoxylin was added and washed after 3 min. Samples were immersed in ethanol 100%, 96%, 80%, 70% and xylol, consecutively. Propidium iodide (PI) was used for background fluorescent removal. Photographs were taken from stained slides, and the number of TUNEL‐positive cells was counted in each image using a LABOMED TCM 400 Fluorescent microscope.

### 2.6 MDA

MDA kit was used to measure the oxidative stress induced by radiation. To assess MDA level in the tongue, specimens were snap‐frozen after collection. The specimens were kept at −80°C until assay. A Biocore Diagnostik (ZellBio) MDA assay Kit was used to measure the lipid peroxidation rate in tissue homogenates. The kit was used according to manufacturer's instructions. Tissue specimens or standard (100 μl) were added to wells. Then, reagent and chromogenic solution were added, respectively. After 1 h of boiling, the mixture was cooled and the absorbance of the supernatant was read at 535 nm. MDA level was measured according to the standard curve.

### 2.7 Griess test

Griess test was used to assess the concentration of nitrite, a product of NO, in the mucosal tissue. Tissue homogenate was prepared, and the supernatant was separated for measuring nitrite level. To measure the tissue level of NO, a Griess reagent assay kit (Sigma‐Aldrich, G4410) was used. First, tissue supernatant and an equal volume of Griess reagents (100 μl) were mixed. After 15 min, the pink colour was metered at 570 nm by a microplate spectrophotometer. Nitrite concentration was calculated against a nitrite standard.

### 2.8 ELISA

TNF‐α, IL1β and IL6 were measured by ELISA. Similar to MDA, tissue samples were snap‐frozen and kept at −80°C until assay. DuoSet®ELISA kits were used for measuring rat TNF‐α, IL1β and IL6. The kits were used following manufacturer instructions. High‐quality Bovine Serum Albumin (BSA) was utilized as the reagent diluent. The sandwich ELISA method was done with rat TNF‐α, IL1β and IL6 capture antibodies and rat TNF‐α, IL1β and IL6 detection antibodies. Standards or tissue homogenates (100 μl) were added to each well and incubated for 2 h at room temperature. After plate preparation, 100 μl of detection antibodies (biotinylated goat anti‐rat TNF‐α detection antibody for TNF‐α, biotinylated goat anti‐rat IL1β detection antibody for IL1β and biotinylated goat anti‐rat IL6 detection antibody for IL6) were added to each well. After 2 h of incubation at room temperature, washing was repeated. Thereafter, 100 μl of the working dilution of streptavidin‐HRP B was added to each well and incubated for 20 min at room temperature. Then, 100 μl of substrate solution was added to each well and incubated for 20 min at room temperature. Eventually, 50 μl of stop solution was added to each well, and their absorbance was read at 450 nm to determine their optical density.

### 2.9 Western blotting

To measure the effect of radiation on the signalling pathways and apoptosis, we used Western blotting. Tongue tissue homogenate was prepared using the following chemicals as the lysis buffer (Tris–HCl (500 μl, PH = 8), EDTA (0.003 g), NaCl (0.08 g), sodium deoxycholate (0.025 g), SDS (0.01 g) and protease inhibitor cocktail (1 tablet) and NP40 1%). The samples were centrifuged (at a speed of 1200 rounds/min, 4°C for 10 min). The supernatants were obtained and kept at −20°C. Bradford protein assay using the Bradford reagent (coomassie blue G250 (5 mg), ethanol 95% (2.5 mg), phosphoric acid (5 mg) and distilled water (50 ml)) was used to assess protein concentration. BSA was used for drawing the standard curve, and 12 μl of samples with similar concentrations was added to each well using a Hamilton syringe. After boiling in 100°C water, the samples were resolved on 10% SDS‐PAGE gel and after the running phase transferred onto polyvinylidene fluoride or polyvinylidene difluoride (PVDF) membranes, using a transfer buffer consisting of tris (2.42 g), glycine (11.25 g), methanol (20 ml) and water (80 ml). Membranes were blocked with tris‐buffered saline with 0.1% Tween (TBST) buffer + skim milk 2% for 75 min at room temperature. Thereafter, membrane was probed with diluted primary antibodies (β‐actin (sc‐47,778, 1: 300), p‐NF‐κB (sc‐136,548, 1:300), NF‐κB (ab‐16,502: 1:300), p‐ERK (sc‐16,981‐R, 1:300), JNK (sc‐7345, 1:300), p‐JNK(sc‐6254, 1:300), bax (sc‐7480, 1:300) and bcl‐2 (sc‐492, 1:300)) for 14–16 h. Membranes were then washed and incubated with secondary antibodies (sc‐2357, 1:1000) for 75 min at room temperature. Then, the membranes were washed with TBST buffer for three times and each time for 15 min. In the next step, membranes were detected with an ECL detection kit by gel doc. An open‐source image processing software, Image J, was used to assess the optical density of each band.

### 2.10 Statistical analysis

Data were analysed by GraphPad Prism version 7. One‐way anova followed by post hoc Tukey's test was utilized for the analysis of data. Differences were interpreted as significant when *p* < 0.05. Charts are shown as mean ± SEM.

## 3 RESULTS

### 3.1 Sildenafil decreased apoptosis of mucosal cells and could partly preserve the integrity of the mucosal barrier against radiotherapy

**FIGURE 1:**
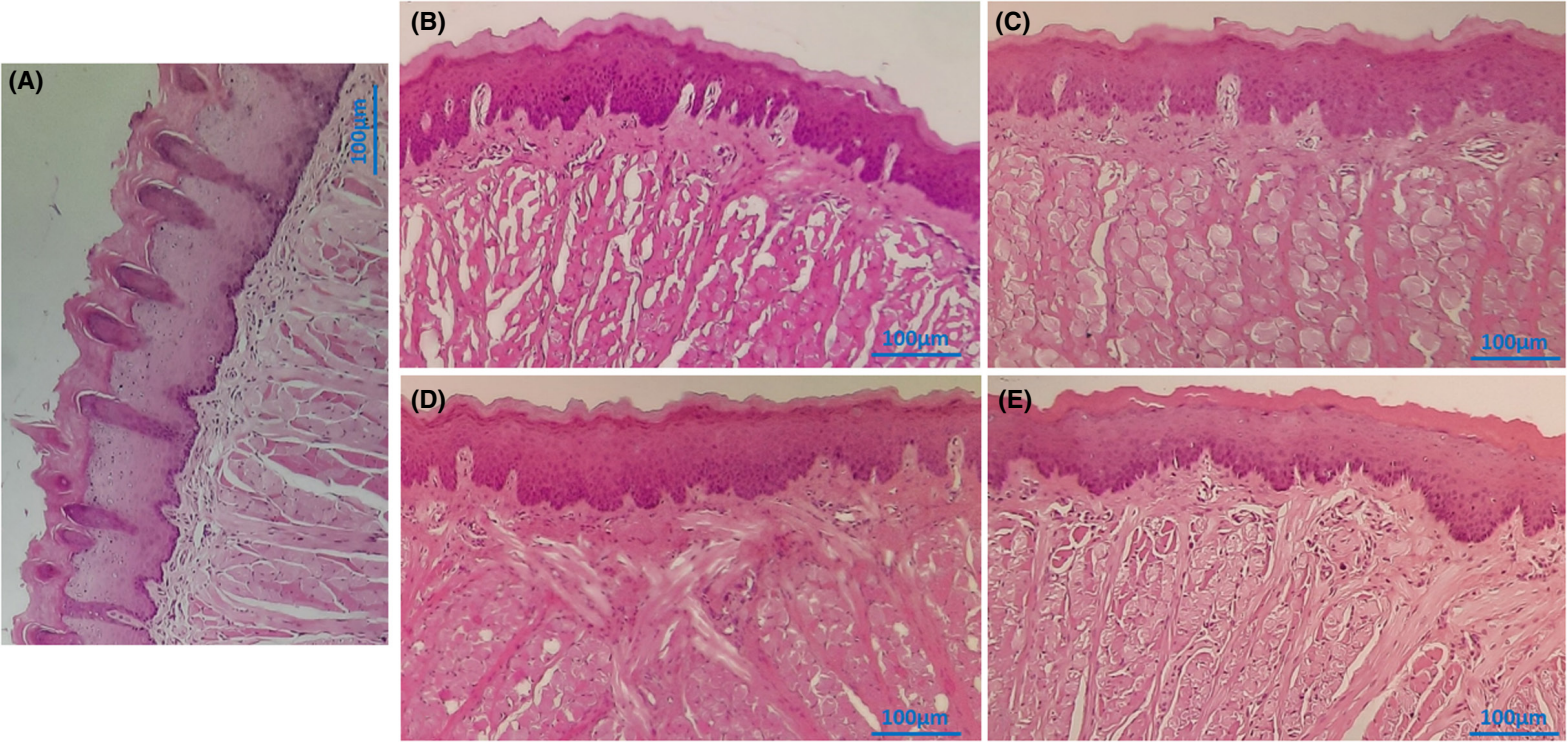
Histopathological changes of tongue mucosal layer after exposure to 8 Gy X‐ray. Low‐dose radiation did not markedly change the mucosal structure in histopathology. Normal control (A), irradiated control (B) and sildenafil‐treated rats [5 (C), 10 (D) and 40 (E)] all had nearly similar mucosal integrity

**FIGURE 2:**
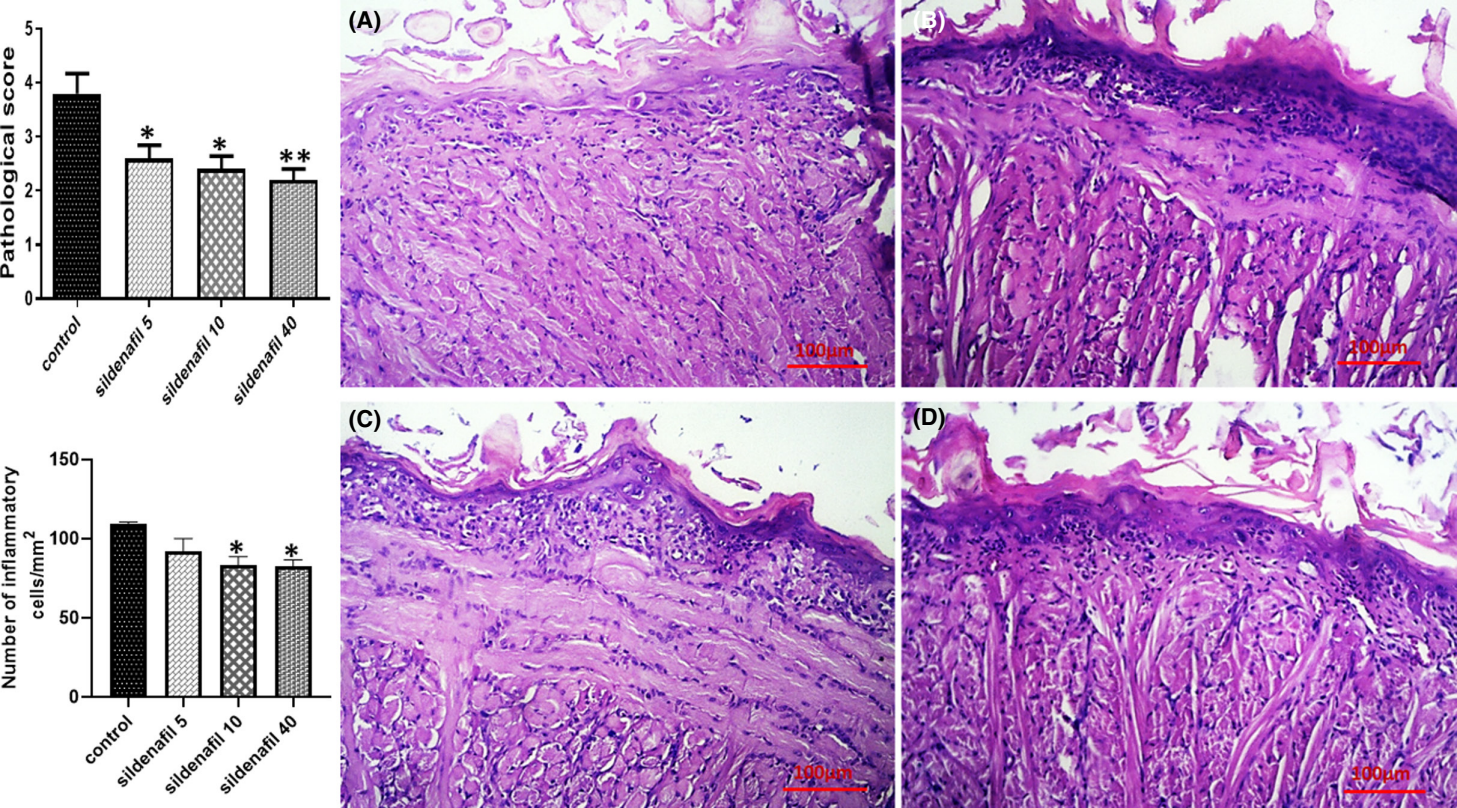
Histopathological changes of tongue mucosal layer after exposure to 26 Gy X‐ray. Irradiation severely distorted tongue mucosal structure in the control group (A) and nearly destructed the mucosal barrier. Sildenafil 5 (B), 10 (C) and 40 (D) mg/kg/day partly preserved the mucosal layer and significantly decreased (*p < 0.05, *, *p* < 0.05 and **p < 0.01, respectively) tissue pathological score. In addition, sildenafil 10 and 40 mg/kg significantly (*p < 0.05) decreased inflammatory cell infiltration

**FIGURE 3:**
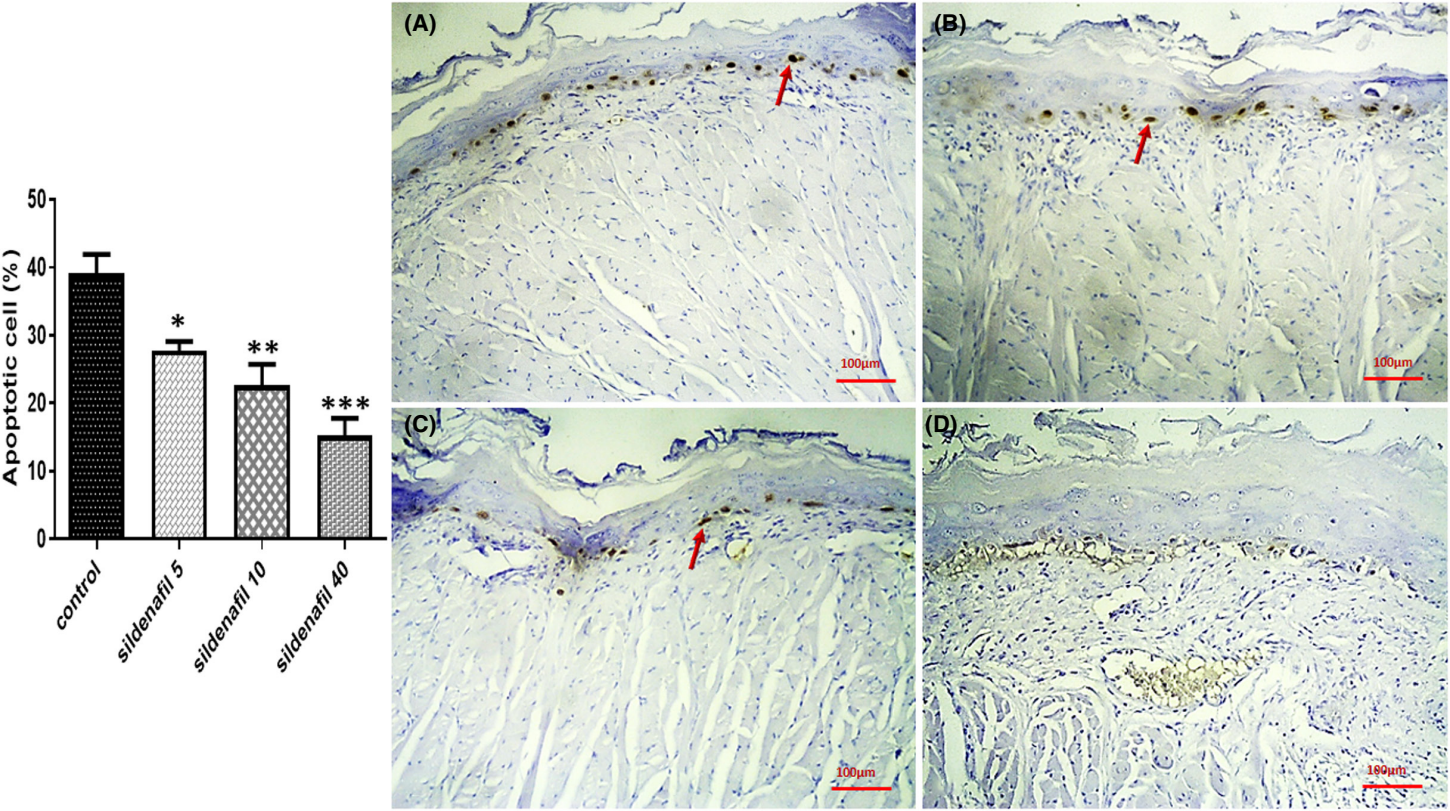
TUNEL assay of the tongue tissue after irradiation with 26 Gy X‐ray. Sildenafil 5 (B), 10 (C) and 40 (D) mg/kg/day could significantly (*p < 0.05, **p < 0.01 and ***p < 0.001, respectively) improve mucosal cells survival and decrease apoptosis, compared with saline‐treated controls (A). Red arrows are indicating apoptotic cells

Rats were sacrificed 7 days after irradiation, and tissue specimens (*n* = 5) were collected. H&E staining was performed, and the histopathological view of specimens was interpreted. It was shown that low‐grade radiation (8 Gy) could not lead to a significant change in histopathology and all groups had nearly the same normal tissue structure (Figure 1). No defect was found in the mucosal layer integrity, and inflammatory cell infiltration into the tissue was negligible. However, high‐grade radiation (26 Gy) severely destructed the mucosal barrier. The mucosal layer was almost absent in some samples of the control group. The use of sildenafil was associated with an improved mucosal structure. Sildenafil 5, 10 and 40 mg/kg significantly (*p < 0.05, *p < 0.05 and **p < 0.01, respectively) decreased pathological score of the tissue (Figure 2) and sildenafil 10 and 40 mg/kg (*p < 0.05) reduced inflammatory cells infiltration (Figure 2). Furthermore, the TUNEL assay revealed a high amount of apoptosis in the control group. Sildenafil 5, 10 and 40 mg/kg significantly (*p < 0.05, **p < 0.01 and ***p < 0.001, respectively) decreased apoptosis of mucosal cells (Figure 3).

### 3.2 Sildenafil significantly decreased the increased level of inflammatory cytokines, nitrite and MDA

**FIGURE 4:**
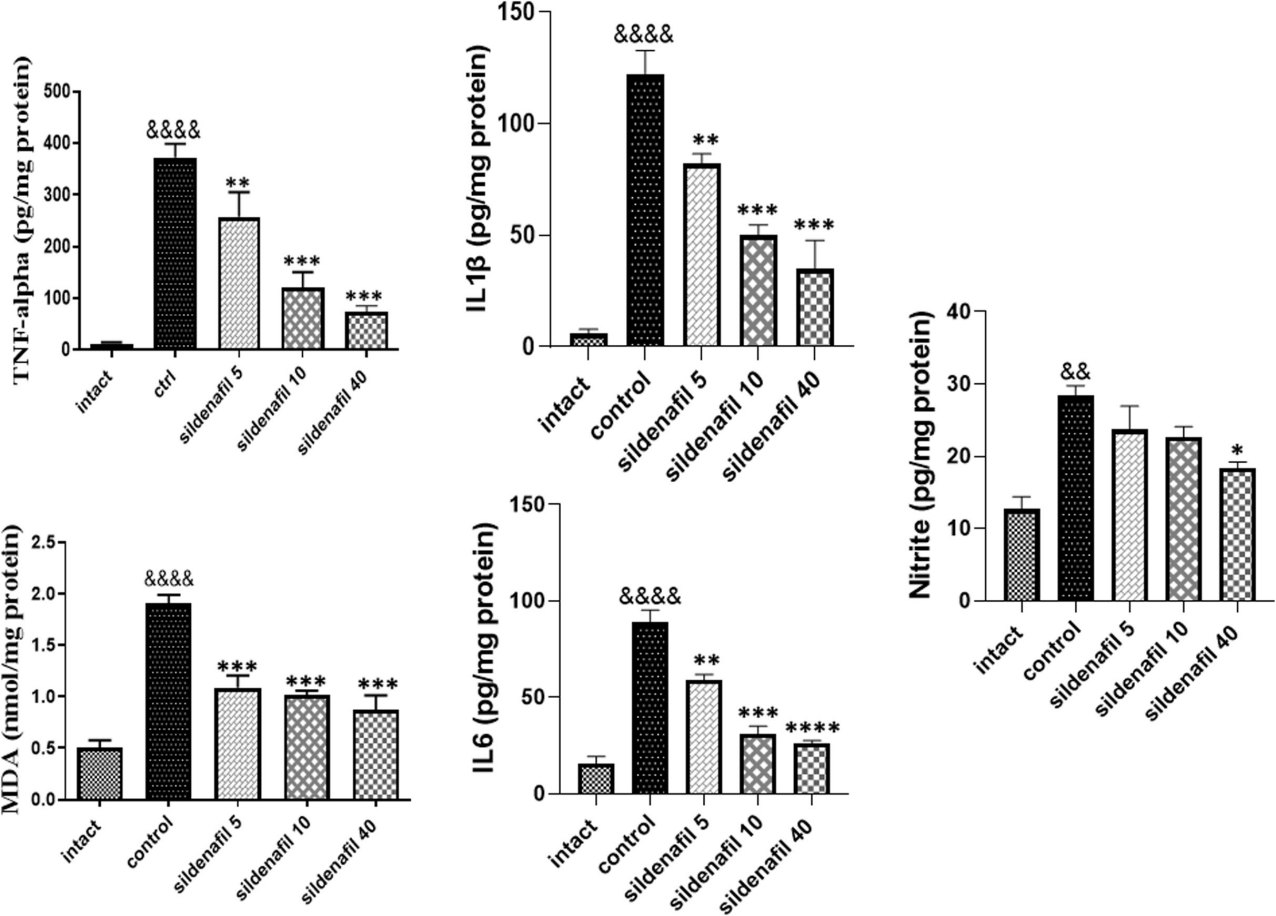
Effects of sildenafil on oxidative stress and inflammatory cytokines. Radiation‐induced oral mucositis led to remarkable (&&&&*p* < 0.0001) increase in IL1β, IL6, TNF‐α and MDA levels. Low‐dose radiation also increased nitrite level (&&*p* < 0.01). Sildenafil significantly decreased MDA (***, *p* < 0.001), TNF‐α (**p < 0.01 for 5 mg/kg/day and ***p < 0.001 for 10 and 40 mg/kg/day), IL1β (**p < 0.01 for 5 mg/kg and ***, *p* < 0.001 for 10 and 40 mg/kg), IL6 (**p < 0.01 for 5 mg/kg and,***, *p* < 0.001 for 10 mg/kg/day and *p* < 0.0001,**** 40 mg/kg/day) and nitrite (*p < 0.05 for 10 mg/kg/day), compared to the control groups

However, low‐dose radiation (8 Gy) showed no significant impact on the histologic view of the mucosal layer (*n* = 5); further measurements were performed to uncover the molecular alterations caused by irradiation and sildenafil in the tissue. We found that low‐dose radiation significantly (&&&&*p* < 0.0001) enhanced the production of inflammatory cytokines including TNF‐α, IL1β, IL6 and increased MDA levels, as a marker of oxidative stress. Low‐dose radiation also significantly (&&*p* < 0.01) increased nitrite level, as measured by the Griess test. In contrast, sildenafil partly reversed these changes and significantly decreased MDA (***p < 0.001), TNF‐α (**p < 0.01 for 5 mg/kg/day and ***p < 0.001 for 10 and 40 mg/kg/day), IL1β (**p < 0.01 for 5 mg/kg/day and ***p < 0.001 for 10 and 40 mg/kg/day), IL6 (**p < 0.01 for 5 mg/kg/day, ***p < 0.001 for 10 mg/kg/day and ****p < 0.0001 for 40 mg/kg/day) and nitrite level (*p < 0.05 for 10 mg/kg/day). Sildenafil 40 mg/kg was the most effective dose in this study regarding its effects on the inflammatory mediators (Figure 4).

### 3.3 Sildenafil decreased the expression and phosphorylation of NF‐κB as well as ERK phosphorylation

**FIGURE 5:**
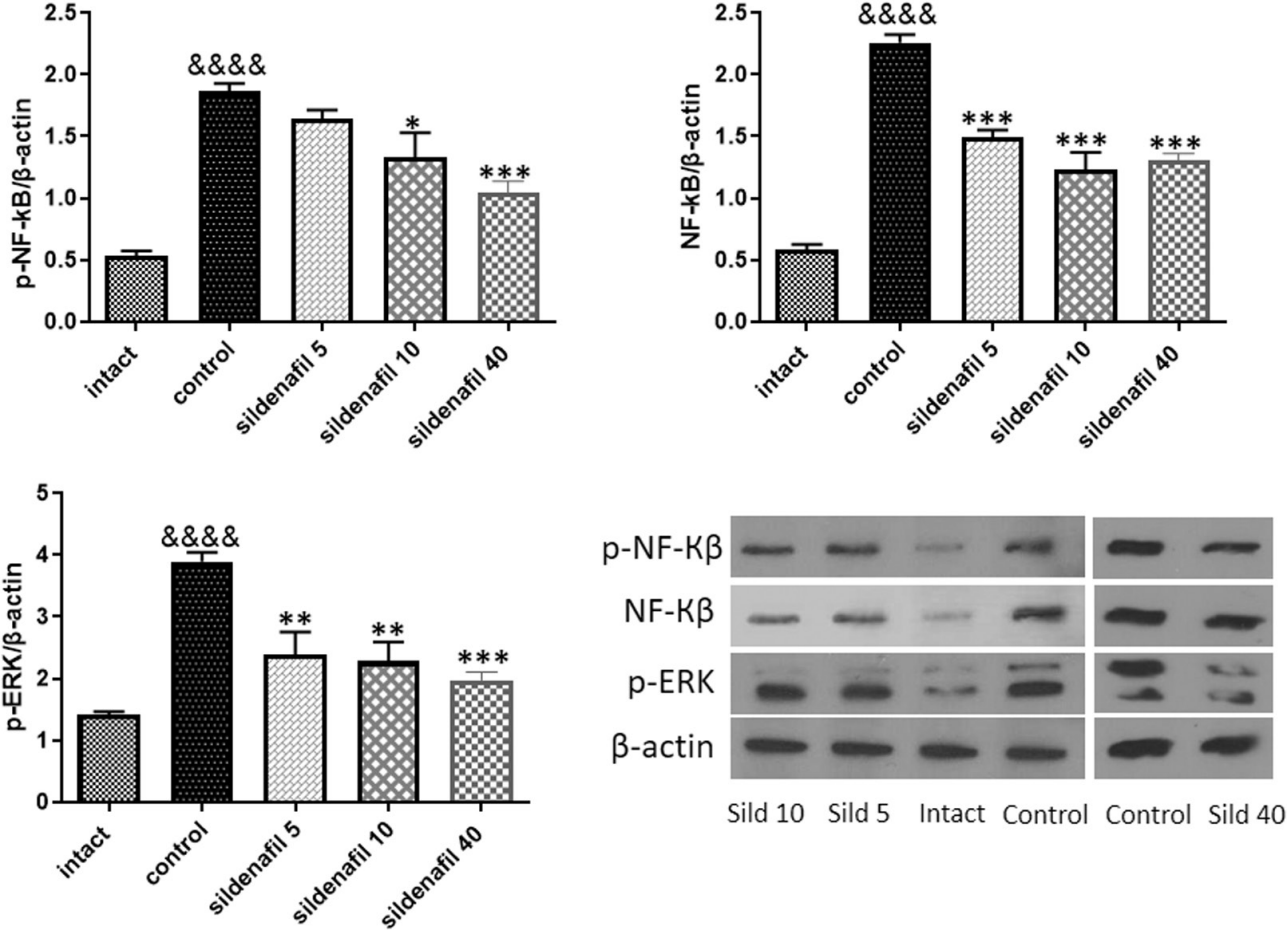
Effects of sildenafil on NF‐κB and ERK signalling pathways. Induction of mucositis was associated with a significant (&&&&*p* < 0.0001) increase in the expression of NF‐κB and its phosphorylation. P‐ERK/β‐Actin markedly (&&&&*p* < 0.0001) increased after irradiation, as well. Treatment with sildenafil was associated with significant decrease in the expression of NF‐κB (***p < 0.001) p65‐NF‐κB/β‐Actin (*p < 0.05 for 10 mg/kg/day and ***p < 0.001 for 40 mg/kg/day) and p‐ERK/β‐Actin (**p < 0.01 for 5 and 10 mg/kg/day and ***p < 0.001 for 40 mg/kg/day). The same band is shown for β‐Actin of the control group and sildenafil 40 mg/kg in Figures 5 and 6 because the protein bands presented for the control group and sildenafil 40 mg/kg in Figures 5 and 6 belong to the same samples

To discover how sildenafil can ameliorate inflammation in radiation‐induced oral mucositis (*n* = 5), we measured the major inflammatory pathways involved in the pathogenesis of oral mucositis. Low‐dose irradiation (8 Gy) led to a significant (&&&&*p* < 0.0001) increase in NF‐κB expression and p65‐NF‐κB/β‐actin ratio as well as p‐ERK/β‐actin ratio. Treatment with sildenafil has been associated with significantly decreased NF‐κB/β‐actin (***p < 0.001), p65‐NF‐κB/β‐actin (*p < 0.05 for 10 mg/kg/day and ***p < 0.001 for 40 mg/kg/day) and p‐ERK/β‐actin (**p < 0.01 for 5 and 10 mg/kg/day and ***p < 0.001 for 40 mg/kg/day) (Figure 5). Sildenafil 40 mg/kg was more effective to downregulate NF‐κB expression, its p65 phosphorylation and ERK phosphorylation.

### 3.4 Sildenafil decreased JNK phosphorylation and increased bcl‐2/bax ratio

**FIGURE 6:**
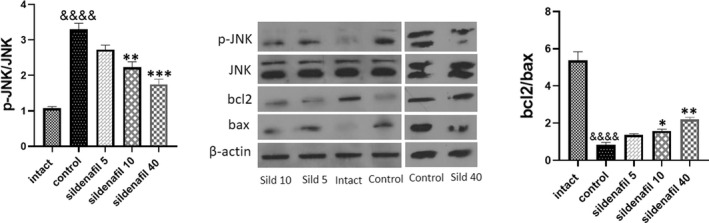
Effects of sildenafil on JNK phosphorylation and bcl2/bax ratio. Irradiation was associated with conceivably increase (&&&&*p* < 0.0001) in the phosphorylation of JNK and decrease in bcl‐2/bax ratio (&&&&*p* < 0.0001) Treatment with sildenafil was associated with significant decrease in p‐JNK/JNK (**p < 0.01 for 10 mg/kg/day and ***p < 0.001 for 40 mg/kg/day). Sildenafil could also increase bcl2/bax ratio (*p < 0.05 for 10 mg/kg/day and **p < 0.01 for 40 mg/kg/day). The same band is shown for β‐Actin of the control group and sildenafil 40 mg/kg in Figures 5 and 6 because the protein bands presented for the control group and sildenafil 40 mg/kg in Figures 5 and 6 belong to the same samples

In addition to the TUNEL assay, we measured the levels of apoptosis markers and activation of the JNK pathway as a major apoptosis pathway in oral mucositis (*n* = 5). Western blotting showed that exposure to low‐dose radiation was associated with a significant increase (&&&&*p* < 0.0001) in p‐JNK/JNK ratio and a significant decrease (&&&&*p* < 0.0001) in bcl‐2/bax ratio. Sildenafil significantly decreased p‐JNK/JNK ratio (**p < 0.01 for 10 mg/kg/day and ***, *p* < 0.001 for 40 mg/kg/day) and increased bcl‐2/bax ratio (*p < 0.05 for 10 mg/kg/day and **p < 0.01 for 40 mg/kg/day) (Figure 6). Similar to the TUNEL assay, sildenafil 40 mg/kg was more effective in preventing apoptosis of oral mucosal cells.

## 4 DISCUSSION

The present study has shown that sildenafil can alleviate inflammation and decrease oxidative stress after head and neck irradiation. Consistently, sildenafil decreased the apoptosis of mucosal cells. In addition, the drug partly preserved mucosal structure after irradiation. The current study has shown that sildenafil partly attenuated oxidative stress, and inhibited NF‐κB, ERK and JNK signalling pathways. The drug significantly decreased inflammatory cytokines including TNF‐α, IL1β, IL6 and nitrite levels. In addition, sildenafil also prevented mucosal cell apoptosis.

Radiotherapy leads to the production of a large amount of ROS, which can cause mucosal injury. The burst of oxidative stress can promote the inflammatory response.^24^ Soon, several inflammatory mediators are produced. DNA breaks and cell damage finally upregulates pro‐apoptotic pathways.^24^ ROS are a major driver for radiotherapy‐induced oral mucositis, and inhibition of mitochondrial ROS production was shown to be protective against post‐radiotherapy oral mucositis.^25^ Consistently, anti‐oxidants could effectively ameliorate radiation‐induced oral mucositis.^26^ The current study showed that sildenafil can significantly decrease the tissue concentration of MDA, a marker of oxidative stress. In addition, sildenafil decreased NO production mainly produced by iNOS (inflammatory nitric oxide) in oral mucositis to augment inflammation.^27^, ^28^ Previously, it was shown that decreased function of iNOS and decreased levels of NO metabolites are associated with a better outcome of oral mucositis.^27^, ^28^ Hence, sildenafil may help to inhibit the initiation of oral mucositis.

It was uncovered that head and neck cancer patients who have undergone chemoradiation therapy have higher salivary levels of inflammatory cytokines such as IL1β, IL6 and TNF‐α.^29^ Meanwhile, higher levels of these cytokines predicted more severe oral mucositis.^29^ Similarly, radiation‐induced oral mucositis is associated with a significant increase in inflammatory cytokines such as IL1β and TNF‐α and alleviation of mucositis led to a significant decrease in inflammatory cytokines levels.^30^ Successful treatment of oral mucositis is associated with a conceivable reduction in the production of inflammatory cytokines such as IL1β and TNF‐α.^31^, ^32^, ^33^ Increased levels of inflammatory cytokines result in the apoptosis of mucosal cells after radiotherapy and endanger the integrity of the mucosal barrier.^34^ Treatment with sildenafil effectively decreased IL1β, IL6 and TNF‐α in this study.

NF‐κB is strongly involved in the progression and amplification of inflammatory response. Its phosphorylation and nuclear translocation are followed by the production of numerous inflammatory cytokines.^35^ ROS can activate NF‐κB to increase the production of inflammatory cytokines.^3^ Hence, the NF‐κB signalling pathway is responsible for amplification of inflammatory response in radiation‐induced oral mucositis.^3^ Downregulation of NF‐κB signalling protects against oral mucositis and is associated with improvement in mucosal histology.^4^, ^36^ Radiation‐induced mucositis is associated with increased ERK phosphorylation.^4^ Indeed, ERK phosphorylation is a sign of inflammation and mucosal damage.^37^, ^38^ Also, decreased ERK phosphorylation is associated with the alleviation of mucositis.^39^ In addition, overactivation of the ERK pathway is involved in cell cycle arrest and apoptotic cell death and attenuation of the ERK signalling pathway may prevent apoptosis.^40^, ^41^ In this study, the protective effects of sildenafil on radiation‐induced oral mucositis have been associated with a significant decrease in the expression of NF‐κB and phosphorylation of NF‐κB and ERK.

Radiation and inflammation enhance the JNK signalling pathway.^42^, ^43^ JNK participates in intrinsic and extrinsic pathways of apoptosis. In response to environmental stress such as radiation, JNK upregulates the expression of pro‐apoptotic proteins.^44^ Even, JNK deletion prevents apoptotic cell death in response to ultraviolet (UV) radiation.^44^ JNK decreases bcl‐2/bax ratio to promote apoptotic cell death.^45^ Sildenafil decreased JNK phosphorylation and increased bcl‐2/bax ratio. As a result, sildenafil‐treated rats showed lower apoptosis in the TUNEL assay.

## 5 CONCLUSION

Taken together, our findings show that sildenafil can improve radiation‐induced oral mucositis and prevent apoptosis of mucosal cells. Its effect on oral mucositis has been associated with attenuation of NF‐κB, ERK and JNK signalling and reduction of oxidative stress and inflammatory cytokines release.

## AUTHOR CONTRIBUTIONS

**Moein Ala:** Conceptualization (equal); data curation (equal); methodology (lead); writing – original draft (lead). **Razieh Mohammad Jafari:** Data curation (equal); investigation (equal); writing – review and editing (equal). **Mahan Ala:** Investigation (equal); project administration (equal). **Sedigheh Marjaneh Hejazi:** Methodology (equal); project administration (equal). **Seyed Mohammad Tavangar:** Methodology (equal); validation (equal). **Seied Rabi Mahdavia:** Methodology (equal); project administration (equal). **Ahmad Reza dehpour:** Conceptualization (equal); supervision (equal); validation (equal).

## CONFLICT OF INTEREST

The authors declare no conflicts of interest.

## Data Availability

Data analyzed for this article will be available from the corresponding author upon a reasonable request.
